# 18例浆母细胞淋巴瘤患者的临床特征及预后

**DOI:** 10.3760/cma.j.cn121090-20241215-00571

**Published:** 2025-09

**Authors:** 珊珊 翁, 晴 施, 维莅 赵, 坚青 糜, 黎 王

**Affiliations:** 1 上海血液学研究所，国家医学基因重点实验室，国家转化医学研究中心，上海交通大学医学院附属瑞金医院血液科，上海 200025 Shanghai Institute of Hematology, National Key Laboratory of Medical Genetics, National Translational Medicine Research Center, Department of Hematology, Ruijin Hospital, Shanghai Jiaotong University School of Medicine, Shanghai 200025, China; 2 温州市中心医院（温州医科大学定理临床学院）血液化疗科，温州 325000 Department of Hematology, the Dingli Clinical College of Wenzhou Medical University, Wenzhou Central Hospital, Wenzhou 325000, China

## Abstract

浆母细胞淋巴瘤（plasmablastic lymphoma，PBL）是一类罕见的高侵袭性非霍奇金淋巴瘤，临床缺乏标准治疗方案推荐。本文回顾性分析上海瑞金医院2012年7月至2024年6月确诊的18例PBL患者临床资料，男12例、女6例，患者中位年龄为59（39～77）岁。Ann Arbor分期为Ⅲ～Ⅳ期12例（66.7％）、伴血细胞减少9例（50.0％）、LDH升高12例（66.7％）、Ki-67指数≥90％ 4例（22.2％）。患者肿瘤细胞高表达CD38（15/17，88.2％）/CD138（12/17，70.6％），B细胞标志物CD20少见（1/17，5.9％）。11例基因测序显示常见突变包括TP53（27.3％）、KMT2D（18.2％）和TET2（18.2％）等。排除1例HIV阳性患者未治疗死亡，17例患者经过一线治疗，完全缓解10例（58.8％）、部分缓解5例（29.4％）。中位随访时间为4.33（0.17～12.17）年，患者总体2年无进展生存率和总生存率分别为（68.5±11.2）％和（75.5±10.1）％。

## Abstract (en)

Plasmablastic lymphoma（PBL）is a rare, highly aggressive non-Hodgkin lymphoma subtype for which no standardized therapeutic regimen has been established in clinical practice. This study retrospectively analyzed 18 PBL cases at Shanghai Ruijin Hospital from July 2012 to June 2024. Participants comprised 12 males and 6 females, with a median age of 59（39–77）years. Twelve（66.7％）cases presented at stage Ⅲ/Ⅳ, nine（50％）have cytopenia, 12（66.7％）have increased lactate dehydrogenase level, and four（22.2％）had a Ki-67 index of ≥90％. The tumor cells highly expressed CD38（15/17, 88.2％）/CD138（12/17, 70.6％）, whereas the B-cell marker CD20 was rarely detected（1/17, 5.9％）. Of the 11 cases that underwent genetic sequencing, common mutations included TP53（27.3％）, KMT2D（18.2％）, and TET2（18.2％）. After excluding one patient with positive HIV who died without treatment, 17 patients received first-line therapy, achieving a complete response in 10（58.8％）and a partial response in 5（29.4％）cases. With the median follow-up time of 4.33（0.17–12.17）years, Kaplan-Meier analysis indicated that the 2-year progression-free survival rate and overall survival rate were（68.5±11.2）％ and（75.5±10.1）％, respectively.

浆母细胞性淋巴瘤（plasmablastic lymphoma，PBL）是一种罕见且侵袭性强的B细胞非霍奇金淋巴瘤（NHL），通常与患者的免疫缺陷状态相关[1]。PBL的实验室特征与浆细胞骨髓瘤、免疫母细胞性大B细胞淋巴瘤相似，需要进行细致的鉴别诊断。同时，PBL也具有其独特的特征，如MYC蛋白的过表达、EB病毒（EBV）感染、HIV感染以及间变性淋巴瘤激酶（ALK）的缺失[1]。PBL发病率低、治疗缓解率低、复发率高、预后不良。目前关于PBL的大规模临床研究较少，多为个案报道或小样本研究；治疗方面，传统的CHOP方案治疗效果有限，PBL缺乏统一治疗标准[2]。本研究回顾性分析上海交通大学医学院附属瑞金医院确诊的18例PBL患者的临床特征、治疗反应及预后，以此深化临床医师对PBL的认识并为临床治疗方案的选择提供参考。

## 病例与方法

1. 病例：回顾性分析2012年7月至2024年6月在上海交通大学医学院附属瑞金医院确诊的18例PBL患者的临床资料，其中HIV阳性1例。所有患者均接受组织活检和免疫组化检查，病理诊断均由病理科医师根据世界卫生组织（WHO）2008年和2016年版诊断标准[3]–[4]进行复核。依据Ann Arbor分期系统进行疾病分期。根据NHL IPI评分[5]将患者划分为低-中低危组（0～2分）和中高-高危组（3～5分）；疗效评估以增强CT或PET-CT检查结果为依据，将患者分为治疗有效组［完全缓解（CR）+部分缓解（PR）］和治疗无效组［疾病稳定（SD）+疾病进展（PD）］[6]。本研究获得上海交通大学医学院附属瑞金医院伦理委员会批准［批件号：2019伦理审第（96）号］，所有入组患者均签署了知情同意书。

2. 治疗情况：18例PBL患者中，1例54岁男性患者分期为ⅠE期，考虑病变局限于胃部，患者仅接受了局部放疗；接受PAD（硼替佐米+阿霉素+地塞米松）/PCD（硼替佐米+环磷酰胺+地塞米松）方案化疗4例；接受V-CHOP（硼替佐米+环磷酰胺+阿霉素+长春地辛+泼尼松）方案化疗9例；接受V-EPOCH（硼替佐米+依托泊苷+泼尼松+长春地辛+环磷酰胺+阿霉素）方案化疗3例。1例HIV阳性患者因并发症在治疗前死亡。2例CR患者进一步行auto-HSCT、1例PR患者行auto-HSCT。

3. 随访：通过查阅病历或电话随访，随访截至2024年8月31日。总生存（OS）期为从确诊到末次随访或患者死亡的时间；无进展生存（PFS）期为从确诊到末次随访或PD或任何原因导致患者死亡的时间。

4. 统计学处理：采用SPSS软件和Graphpad prism 8进行统计分析。计量资料以*M*（范围）或均值±标准误进行统计学描述；计数资料以频数（百分比）、例（％）表示；生存分析采用Kaplan-Meier法绘制生存曲线。分类变量间采用Fisher精确检验法进行比较。*P*<0.05认为差异具有统计学意义；鉴于样本量较小，*P*<0.2亦认为有一定的趋势。

## 结果

1. 临床特征：18例PBL患者中，男12例、女6例，中位发病年龄为59（39～77）岁，其中1例为HIV阳性。起病部位以结外病灶为首发症状的有13例，包括胃肠道（7例）、肌肉及周围软组织（1例）、鼻咽（1例）、肝（1例）、睾丸（1例）、扁桃体（1例）和腮腺（1例）；淋巴结肿大起病5例。Ann Arbor分期Ⅰ～Ⅱ期6例、Ⅲ～Ⅳ期12例；LDH升高12例；IPI评分显示，低-中低危组12例、中高-高危组6例。外周血EBV-DNA阳性5例（27.8％）、阴性13例（72.2％）。EBV编码的小RNA（EBER）阳性8例（44.4％）、阴性10例（55.6％）。8例PBL患者检测人类疱疹病毒8型（HHV-8），结果均为阴性。9例PBL患者起病时血细胞一系或两系减少，6例患者既往有肿瘤病史或长期使用免疫抑制剂。此外，10例患者伴有合并症，如糖尿病等（表1）。

**表1:** 18例浆母细胞淋巴瘤患者的临床特征［例（％）］

临床特征	总体（18例）	治疗有效^a^组（15例）	治疗无效^b^组（3例）
年龄			
≤60岁	11（61.1）	9（81.8）	2（18.2）
>60岁	7（38.9）	6（85.7）	1（14.3）
性别			
男	12（66.7）	11（91.7）	1（8.3）
女	6（33.3）	4（66.7）	2（33.3）
Ann Arbor分期			
Ⅰ～Ⅱ	6（33.3）	5（83.3）	1（16.7）
Ⅲ～Ⅳ	12（66.7）	10（83.3）	2（16.7）
LDH			
升高	12（66.7）	9（75.0）	3（25.0）
正常	6（33.3）	6（100）	0（0）
IPI评分（分）			
0～2	12（66.7）	10（83.3）	2（16.7）
3～5	6（33.3）	5（83.3）	1（16.7）
EBV-DNA			
阳性	5（27.8）	3（60.0）	2（40.0）
阴性	13（72.2）	12（92.3）	1（7.7）
免疫抑制状态^c^			
有	6（33.3）	5（83.3）	1（16.7）
无	12（66.7）	10（83.3）	2（16.7）
EBER			
阳性	8（44.4）	6（75.0）	2（25.0）
阴性	10（55.6）	9（90.0）	1（10.0）
Ki-67指数			
≥90％	4（22.2）	2（50.0）	2（50.0）
<90％	14（77.8）	13（92.8）	1（7.2）
血细胞数值			
正常	9（50.0）	9（100）	0（0）
一系或两系减少	9（50.0）	6（66.7）	3（33.3）

**注** ^a^治疗有效：完全缓解+部分缓解；^b^治疗无效：疾病稳定+疾病进展；EBV：EB病毒；EBER：EB病毒编码的小RNA；^c^表示有肿瘤病史或长期使用免疫抑制剂

2. 病理特征：肿瘤细胞广泛表达浆细胞标志物，CD38阳性率为88.2％（15/17），其次是c-Myc（75％，12/16）和CD138（70.6％，12/17）；CD79a阳性率为64.7％（11/17），PD-L1阳性率为41.7％（5/12）；CD10、CD30、Bcl-2、Bcl-6阳性率较低，分别为33.3％（6/18）、23.5％（4/17）、22.2％（4/18）和16.7％（3/18）；B细胞标志物CD20阳性率最低，仅为5.9％（1/17）；EBER阳性率为44.4％（8/18）。

3. 基因突变：11例患者进行了与淋巴瘤预后相关的55个基因的靶向测序，结果如图1所示，突变频率>10％的基因包括TP53（3/11，27.3％）、KMT2C（2/11，18.2％）、KMT2D（2/11，18.2％）、TET2（2/11，18.2％）、IGLL5（2/11，18.2％）、DUSP2（2/11，18.2％）、ITPKB（2/11，18.2％）。

**图1:**
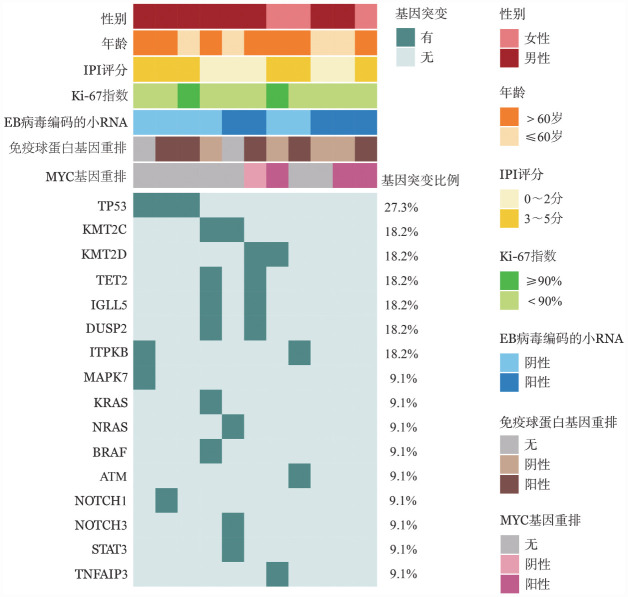
11例浆母细胞淋巴瘤患者的基因突变结果

4. 疗效与生存情况：排除1例HIV阳性患者未治疗死亡，17例患者经过一线治疗，CR 10例（58.8％）、PR 5例（29.4％）、PD 2例（11.8％）。2例CR患者行auto-HSCT，目前处于无PD的状态，1例PR患者行auto-HSCT，目前为移植后的恢复中，截至2024年8月31日，处于CR状态。PAD/PCD方案、V-CHOP方案、V-EPOCH方案的治疗有效率分别为75.0％（3/4），88.9％（8/9）和100％（3/3）。因KMT2C和KMT2D均为组蛋白甲基转移酶家族，予合并分析。将突变率>20％的基因（TP53和KMT2C/D）纳入疗效分析，采用Fisher精确检验法比较治疗有效和治疗无效患者的临床特征和病理、分子生物学特点，结果显示，外周血EBV-DNA阳性（*P*＝0.172）、Ki-67≥90％（*P*＝0.108）以及KMT2C/D基因突变（*P*＝0.109）患者的治疗有效率有降低趋势。

18例患者中位随访时间为4.33（0.17～12.17）年，共有6例患者死亡。患者总体中位PFS期和OS期未达到，2年PFS率和OS率分别为（68.5±11.2）％、（75.5±10.1）％。CR患者5年PFS率和OS率均为（85.7±11.1）％。治疗有效（CR+PR）患者中移植与未移植组的中位PFS期和OS期均未达到，2年PFS率分别为（100±0）％和（80.0±12.0）％，2年OS率分别为（100.0±0）％和（90.9±9.1）％。接受PAD/PCD方案治疗的患者2年PFS率和OS率均为（75.0±21.5）％，V-CHOP方案2年PFS率和OS率分别为（58.3±16.4）％和（77.8±14.7）％，V-EPOCH方案治疗2年PFS率和OS率均为（100±0）％。

## 讨论

PBL是NHL的罕见亚型，好发于中老年男性，预后差，中位OS期为6～19个月，3年OS率约25％[7]。PBL常以结外病变为首发症状，常见于口腔和胃肠道，确诊时已处于晚期，40％伴B症状[8]。本研究中，患者中位发病年龄59岁，男性占66.7％，72.2％的患者结外受累，66.7％诊断时已为Ⅲ～Ⅳ期。大多数PBL患者存在免疫抑制状况，尽管PBL与HIV感染有很强的相关性，但国内报道多以HIV阴性为主。本研究18例患者中，只有1例HIV阳性，可能与局部地区的HIV感染率和流行率相关。此外，有研究指出PBL可能与EBV、HHV-8等感染相关[9]，Bailly等[10]报道超过65％的PBL患者EBV阳性。EBV可以改变肿瘤的免疫微环境，包括上调患者T细胞的程序性死亡受体1（PD-1）、细胞毒性T淋巴细胞相关蛋白4（CTLA-4）以及CD38、CD30等分子表达水平，参与诱导肿瘤细胞免疫逃逸。EBV可作为评估疗效和预后的重要生物指标[11]。本研究的18例患者中，EBV-DNA阳性5例（27.8％）、EBER阳性8例（44.4％），EBV-DNA阳性患者治疗有效率较低。

PBL细胞通常呈弥漫性排列，形状呈圆形或椭圆形，胞质丰富，细胞核偏心，中央核仁突出，核分裂象常见，形成“星空”样外观。这些细胞源自浆母细胞，免疫表型与浆细胞肿瘤相似[12]。常见的阳性标志物包括CD79a、MUM-1、CD38和CD138，Ki-67指数高，B细胞标志物如PAX-5、CD19和CD20则较为罕见[13]。本研究PBL细胞广泛表达浆细胞标志物CD38（88.2％），CD138的阳性表达率为70.6％、c-Myc的阳性率为75％、CD79a的阳性率为64.7％；CD20仅在1例中表达，与文献报道相符。Ki-67指数中位数为72％（40％～99％），与PBL不良预后相关[14]，本研究中有22.2％的患者Ki-67指数≥90％，治疗缓解率为50％，2年PFS率和OS率分别为37.5％和33.3％；表明肿瘤生长迅速且恶性程度高，预后不良，与以往研究结果相似。

KMT2基因家族包含多个成员，它们在不同的癌症类型中显示出不同的突变频率[15]。KMT2C和KMT2D为组蛋白H3K4甲基转移酶，催化哺乳动物组蛋白H3K4甲基化，从而调控基因表达[16]，其缺失会导致DNA损伤应答（DDR）和DNA修复相关基因的表达下调，从而促进B细胞淋巴瘤形成；其表达水平和基因突变状态对淋巴瘤患者的预后和治疗反应具有重要影响[17]。本研究对11例患者进行了靶向基因测序，结果显示，KMT2C和KMT2D基因的突变频率均为18.2％。由于其属于同一基因家族，并且考虑到样本数量有限，我们选择将这两个基因的突变情况合并进行分析，KMT2C/D基因的突变频率合计为36.4％。此外，我们发现KMT2C/D基因可能对治疗有效率有一定的影响，与既往文献结果一致[18]。

PBL具有进展快、复发率及病死率高、预后差等特点，传统CHOP方案疗效有限。Makady等[19]发现CHOP方案和EPOCH方案的CR率分别为39.0％和47.0％，总反应率分别为69.0％和78.9％。基于PBL的浆细胞免疫表型特征，蛋白酶体抑制剂硼替佐米已被尝试用于该病的治疗。一项回顾性研究中，16例患者接受4至6个周期的V-EPOCH方案伴或不伴放疗，15例患者（94％）达到缓解，中位随访48个月，中位OS期为62个月，5年OS率为63％[20]。卡非佐米（carfilzomib）是第二代不可逆蛋白酶体抑制剂，具有更低的神经毒性，在浆细胞白血病治疗上获得较好的疗效[21]，而在PBL治疗中尚未报道，有待今后更多的尝试。auto-HSCT无论是作为一线巩固还是挽救治疗，都能显著改善PBL患者的预后[22]。PBL患者诱导化疗CR后行auto-HSCT，移植后2年复发率为30％，2年OS率为53％[19]。本研究16例患者接受硼替佐米联合化疗方案治疗，其中13例达CR，3例在治疗后2个月内出现疾病进展。PAD/PCD方案和V-CHOP方案疗效相似，而V-EPOCH方案的疗效有更优的趋势。本研究例数较少，但结果显示硼替佐米联合高强度EPOCH方案有望改善年轻PBL患者的疗效，与既往研究结果相符。本研究2例化疗后CR的患者行auto-HSCT，至随访结束时仍处于无疾病进展的状态，结果与既往报道结果一致[22]，接受auto-HSCT巩固治疗可进一步改善患者的生存、减少复发，提示auto-HSCT是化疗有效的年轻患者重要治疗选择。

CD38单抗（如达雷妥尤单抗和isatuximab）通过多种机制诱导肿瘤细胞死亡，在多发性骨髓瘤的治疗中表现出显著的疗效，在PBL中也有一定的应用前景[23]。PBL细胞通常高表达CD38，使得CD38单抗成为治疗PBL的理想靶点。在一项复发的PBL患者小样本研究[24]中，3例接受一线达雷妥尤单抗联合EPOCH方案，1例接受达雷妥尤单抗联合来那度胺、地塞米松和阿霉素治疗。4例患者均达到CR，其中3例患者维持缓解状态超过15个月[24]。这些初步结果令人鼓舞，值得在前瞻性试验中进一步评估。本研究PBL患者CD38阳性率高达88.2％，该结果为今后达雷妥尤单抗临床治疗浆母细胞淋巴瘤提供了客观依据。

综上，本研究初步分析了PBL患者的临床特点，发现采用联合硼替佐米的高强度EPOCH方案治疗和早期进行auto-HSCT可能提高疗效。本研究的局限性在于样本量较小，且为回顾性研究，HIV和HHV-8阳性率低，无法研究这些病毒感染对患者疗效和预后的影响。未来仍需大规模多中心的前瞻性研究进一步验证。
